# Using QALYs in telehealth evaluations: a systematic review of methodology and transparency

**DOI:** 10.1186/1472-6963-14-332

**Published:** 2014-08-03

**Authors:** Trine S Bergmo

**Affiliations:** 1 Norwegian Centre for Telemedicine and Integrated Care, University Hospital of North Norway, N-9038 Tromsø Norway

**Keywords:** Telehealth, Telemedicine, Videoconferencing, Remote consultations, E-Health, Health-related quality of life, Quality-adjusted life-years, Cost-utility analysis

## Abstract

**Background:**

The quality-adjusted life-year (QALY) is a recognised outcome measure in health economic evaluations. QALY incorporates individual preferences and identifies health gains by combining mortality and morbidity into one single index number. A literature review was conducted to examine and discuss the use of QALYs to measure outcomes in telehealth evaluations.

**Methods:**

Evaluations were identified via a literature search in all relevant databases. Only economic evaluations measuring both costs and QALYs using primary patient level data of two or more alternatives were included.

**Results:**

A total of 17 economic evaluations estimating QALYs were identified. All evaluations used validated generic health related-quality of life (HRQoL) instruments to describe health states. They used accepted methods for transforming the quality scores into utility values. The methodology used varied between the evaluations. The evaluations used four different preference measures (EQ-5D, SF-6D, QWB and HUI3), and utility scores were elicited from the general population. Most studies reported the methodology used in calculating QALYs. The evaluations were less transparent in reporting utility weights at different time points and variability around utilities and QALYs. Few made adjustments for differences in baseline utilities. The QALYs gained in the reviewed evaluations varied from 0.001 to 0.118 in implying a small but positive effect of telehealth intervention on patient’s health. The evaluations reported mixed cost-effectiveness results.

**Conclusion:**

The use of QALYs in telehealth evaluations has increased over the last few years. Different methodologies and utility measures have been used to calculate QALYs. A more harmonised methodology and utility measure is needed to ensure comparability across telehealth evaluations.

## Background

The outcomes of telehealth interventions are not easily defined, identified or measured [1]. Effectiveness has been measured in a number of different ways, ranging from the impact on processes to the final outcomes. In economic analyses, the measured outcomes have been diagnostic accuracy, avoided travel and reduced hospitalisation. Disease-specific scale measures such as blood glucose levels, reduction in wound size, anxiety and pain levels and quality of life measures have also been used [2-4]. Disease-specific measures are acceptable for assessing technical efficiency, i.e., how to produce a given level of health outcome for the least cost. For example, diabetes-specific measures can be used to assess whether a new technological device is more effective than existing technology in reducing and stabilising blood glucose levels. Disease-specific and quality of life measures do not include the duration of the improvement, nor can they be used to compare costs and outcomes across disease areas. It can be difficult to interpret cost-effectiveness in terms of a specific cost per reduction in blood glucose level. Furthermore, scores obtained from quality of life questionnaires such as the SF-36 Health Survey cannot be used directly in economic evaluations because the scores do not rank health states according to patients’ preferences and are not measured on a death-full health scale.

Consistency in the outcome measures has important implications for the usefulness of cost-effectiveness results in decision making [5]. To aid resource allocation, we need a common metric that enables the comparison of different kinds of improvements across disease areas and can be compared to the costs in a meaningful way. Quality-adjusted life-year (QALY) is one such measure. QALYs were developed to compare health gains; they are recognised as the primary metric to measure health status in economic evaluation [6-9]. QALYs include mortality and morbidity in one single measure [10]. QALYs are the years lived weighted by the quality of life in that time [8]. Comparing costs and QALYs is also known as cost-utility analysis (CUA). The cost-utility framework is constrained to production decisions, i.e., where a decision maker considers how to best allocate an existing budget. In this situation, the objective is often to establish which alternative maximises the health outcome for a given cost. CUA implicitly assumes that one of the programmes will be undertaken regardless of its net benefit [9]. If the decision maker is considering whether it is worthwhile to achieve a particular goal or expand the budget, a broader cost-benefit analysis is needed. Cost-benefit analysis measures all consequences in a monetary unit and addresses the right mixture of healthcare programmes to maximise the health of a society [11].

The literature contains a large number of telehealth reviews [12]. Most economic evaluations in telehealth to date have used a cost-consequence framework or cost-minimisation analysis (CMA) [2,3,13]. A cost-consequence framework lists all benefits alongside costs without synthesising costs and benefits, which can make it difficult to decide whether the intervention produces good value for money. CMA assumes no difference in outcome and compares only the costs. CMA is generally not viewed an appropriate method of analysis in prospective evaluations [14]. However, the purpose of telehealth might be to provide consultations or episodes of care. If the objective is to establish the least costly mode of delivering specific health services, CMA can be a useful framework.

Few economic evaluations of telehealth interventions have measured health gains in QALYs [4]. A review from 2009 found four evaluations measuring QALYs [3]. A more recent review found seven [2]. None of these previous reviews have examined and discussed the way in which QALYs have been calculated and reported in the literature. The estimation of QALYs in telehealth evaluations should be methodologically appropriate, and its reporting should be transparent.

The aim of this paper is to review and discuss the use of QALYs in economic evaluations of telehealth interventions. In particular, this work examines the ways in which health utility data are used to generate QALYs. It also assesses the transparency of the methods used. This paper contributes to the literature in the following ways: (1) it provides an overview of telehealth studies using QALYs within a cost-effectiveness framework, (2) it reports on the methods used in calculating QALYs, (3) it addresses the transparency of the QALY estimation and reporting of results and (4) it discusses the use of QALYs in telehealth evaluations.

### Estimating QALYs

QALYs are estimated in three steps. The first step is to collect preference-based health-related quality of life (HR-QoL) measures to develop health states. HR-QoL measures can be obtained using generic pre-scored descriptive classification systems. One of the most commonly used descriptive systems is the EuroQol-5D (EQ-5D), which was developed by the EuroQol group [15]. EQ-5D is a recognised tool to describe different health states and is recommended in economic evaluation guidelines [9,10,16]. The EQ-5D has five attributes: mobility, self-care, usual activities, pain/discomfort and anxiety/depression, each of which has three levels. Another descriptive system used to derive HRQoL measures is the SF-6D which can be extracted from SF-36 and SF-12 Health Surveys. Brazier and his colleagues simplified these into six dimensions, obtained preference scores and estimated preference weights from the general population using the standard gamble technique [17,18]. The six dimensions are physical functioning, role limitation, social functioning, pain, mental health and vitality, each of which has four to six levels. Other generic pre-scored health state classification systems are the Health Utility Index (HUI) [9], Quality of Well-Being (QWB) [19], Assessment of Quality of Life (AQoL) [20] and 15D [21].

The second step is to attach preference weights (values or utilities) to the different HRQoL measures defined by the descriptive systems. This process involves weighting the relative importance of the different aspects in the questionnaire using preference scores [5]. These are derived from the general population and fall on a scale from 1 (full health) to 0 (death). It is possible to be in a health state worse than death with a negative quality index. The National Institute for Health and Clinical Excellence (NICE) in the United Kingdom recommends a set of values estimated from 3,000 members of the UK population using the time trade-off technique [22]. Other countries have estimated similar country-specific health state utility values [23]. An alternative approach is to ask the patients directly in interviews to describe and value their health status using complex techniques such as time trade-off (a choice between quality of life and longevity of life) or standard gamble (a choice between a certain outcome and a gamble on either better or worse health) [24]. These techniques are more time consuming and expensive than using the population based utility weights.

The third step is to calculate the QALYs gained by including time. This involves multiplying the quality weights for the health states developed in step two with the duration of each health state experienced by the patients. For example, one year in full health is one QALY. Four years in a 0.5 quality state is two QALYs. The general formula for a QALY gain can be written as follows:

$$\mathrm{QALY}\;\mathrm{gain}\;=\;Q_{1}x\;T_{1}–\;Q_{0}x\;T_{0}$$

where Q_1_ x T_1_ refers to the quality weight Q_1_ multiplied by the expected duration T_1_ (expected health status) with intervention or treatment. Q_0_ x T_0_ refers to the quality weight Q_0_ multiplied by the expected duration T_0_ for the usual care or no-treatment alternative.

When costs and QALYs have been measured and valued, the next step is to compare the costs and QALYs of the new intervention to those of the alternative or existing technology on an ordinal level [10]. If the new intervention costs less and generates more QALYs than the existing alternative, then the new technology is cost effective and no further analysis is needed. Similarly, if the services generate less benefit at increased cost, then no further analysis is needed. If the new intervention costs more and is more effective, a more rigorous economic evaluation is needed. In the latter situation, it is necessary to calculate the cost per QALY or the incremental cost-effectiveness ratio (ICER). ICER establishes how much more the new technology costs and how much more effective it is compared to the alternative.

## Methods

The review was limited to economic evaluations of the use of any type of information and communication technology to examine, treat, monitor, follow up or care for patients over a distance, where the outcomes have been measured in QALYs. The interventions evaluated used telemonitoring, store-and-forward transmissions of data, video links, email consultations or structured telephone support. Web-based motivational self-help interventions without any communication with health providers were excluded.

The search was limited to articles written in English and published in peer-reviewed journals between 1990 and 2012. The articles included were economic evaluations, i.e., they undertook a comparative analysis of both costs as resource use and outcomes in the form of QALYs of at least two alternatives. Only cost-effectiveness analyses using primary patient-level data were included. Evaluations using models to extrapolate primary data beyond the trial period were also included. Evaluations synthesising secondary data from a number of different sources into a decision modelling framework, protocol papers describing ongoing evaluations, and evaluations using scores from the descriptive systems or utility data without calculating QALYs were excluded.

The search strategy included two main search terms:

1. (“telemedicine” [MeSH Terms] OR “telehealth” [All Fields] OR telemonitoring

[All Fields] OR telecare [All Fields] OR “remote consultation” [MeSH Terms] OR

teleconsultations [All Fields] OR e-health [All Fields] OR “videoconferencing” [MeSH Terms] OR “telephone” [MeSH Terms] OR Internet- based [All Fields] OR “Internet” [MeSH Terms]) AND

2. (“quality-adjusted life-years” [MeSH Terms] OR “qalys” [All Fields] OR cost-utility [All Fields])

**Figure 1:**
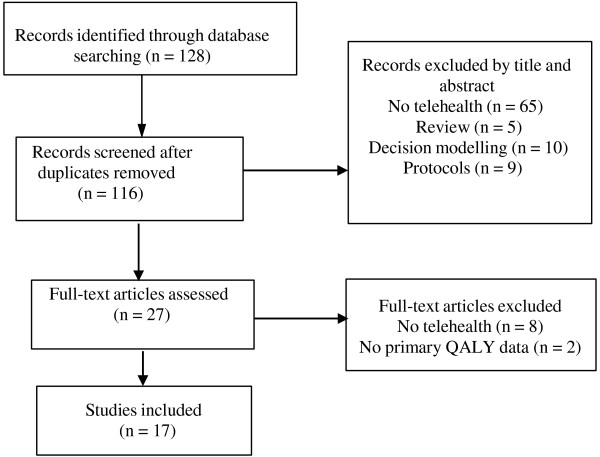
Flow chart of study inclusion.

The electronic literature databases PubMed, PsycInfo and CINAHL were searched using a combination of the search strategy above. The National Health Service Economic Evaluation Database (NSH EED) was searched using “telemedicine” OR “telehealth” OR “videoconferencing” OR “telephone”. The two main journals in the telemedicine field, *Journal of Telemedicine and Telecare* and *Telemedicine Journal and E*-*health*, were searched electronically using only “QALYs” or “cost-utility”.The selection of relevant publications was based on information found in the abstracts. Full-text articles were retrieved when the abstract indicated analyses of both costs and QALYs. Full-text articles were also retrieved for closer inspection if the abstract did not provide a clear indication of the content. All abstracts and full-text articles were read by the author. Figure 1 shows a flow diagram mapping the number of studies identified, included and excluded, as well as the reasons for exclusion.

Information divided into three main categories was extracted and used to assess the reviewed articles. These main categories were as follows: (1) general characteristics of the evaluations, (2) methodology and transparency of the QALY estimation and (3) reporting of results, including handling of uncertainty. Details extracted from the evaluations were as follows: type of intervention, technology used, sample size, effectiveness data, utility values, data collection intervals, costing method, methods for handling uncertainty, how the difference in costs and QALYs was reported, whether incremental cost per QALY was calculated, and key findings.

## Results

### General characteristics

The search strategy described above identified 17 economic evaluations of telehealth interventions. Table 1 provides a summary of the evaluations included in the review. Most evaluations analysed structured telephone support and monitoring as part of a remote follow-up regimen after treatment. Several evaluations analysed a combination of Internet interventions and telephone follow-up. Two evaluations included video link as part of the telehealth follow-up intervention. The papers were published between 2007 and 2012. Most papers were published over the last two years (see Table 2). Seven originated from the Netherlands, five from the United States, two from Australia, two from the United Kingdom and one from Sweden.

**Table 1:** Summary of the economic evaluations

**Article**	**Intervention sample size (N)**	**Utility data**	**Utility intervals**	**Costing**	**Difference in costs and QALYs**	**Key findings (ICER and cost effectiveness results)**
Barnett 2007 [25] USA	Messaging and video for diabetes N = 370	SF-6D (SF-36)	Baseline and 12 months	Health provider, no cost information provided	Difference in QALYs or costs not reported	$60,940 per QALY and cost effective, 23% cost effective at $20 0000, 37% at $100,000 and 42% at a threshold of $200,000
Blankers 2012 [28] Netherland	Internet therapy for harmful alcohol use N = 136	EQ-5D	Baseline and 6 months	Health provider and societal, comprehensive	0.06 QALYs gained, CI/p not reported, increased costs (NS)	Median cost per QALY €14,710. The intervention had a 60% likelihood of being cost effective at threshold €20,000
Franzen 2009 [26] Sweden	Telephone follow-up for injured road users N = 510	EQ-5D	Baseline, (3)^§^, and 6 months	Intervention costs for the health system	0.01 QALYs gained, increased costs, CI/p not reported	42,500 SEK per QALY and cost effective
Graves 2009 [31] Australia	Telephone intervention for physical activity N = 431	SF-6D (SF-36)	Baseline, 4 and 12 months	Health provider, comprehensive	QALYs gained not explicitly reported on individual level*, increased costs	Telehealth vs usual care $78,489 per QALY and not cost effective. Telehealth vs real control (no follow up) $29 375 per QALY and cost-effective
Graves 2009 [32] Australia	Telephone support to prevent re-hospitalisation N = 122	EQ-5D mapped from SF-12	Baseline 4, 12, and 24 weeks	Health provider, comprehensive	0.118 QALYs gained (S), reduced costs NS	NMB $7,907. 100% probability of increased QALYs and 64% probability of reduced costs
Handley 2008 [47] USA	Telephone follow-up in diabetes care N = 226	SF-6D (SF-12)	Baseline and 12 months	Intervention costs for the health provider	0.012 QALYs gained, increased costs, CI/p not reported	$65,167 per QALY gained and within accepted cost effective range without specification
Herbert 2008 [33] USA	Telephone follow-up in heart failure N = 406	HUI3 and EQ-5D mapped from SF-12	Baseline, 3, 6, 9 and 12 months	Societal and payer, comprehensive	0.0497 QALYs gained (HUI3) 0.0430 QALYs gained (EQ-5D) (S), no difference in costs	$17,543 (EQ-5D) and, $15,169 (HUI3) with a 64% and 77% probabilities of cost-effectiveness
Kimman 2011 [42] Netherland	Telephone follow-up after breast cancer treatment N = 299	EQ-5D	Baseline, 3, 6, and 12 months	Societal, comprehensive	QALY gained not reported, increased costs	Telephone the preferred strategy. At a threshold of €80,000, 62% probability of being cost effective
Moss-Morris 2012 [27] UK	Internet and telephone follow-up for fatigue N = 40	EQ-5D	Baseline and 10 weeks	Health provider	0.015 QALYs gained (S), no difference in costs	The intervention is cost-effective
Neelemaat 2012 [43] Netherland	Telephone support to malnourished elderly N = 210	EQ-5D	Baseline and 3 months	Societal, comprehensive	0.02 QALYs gained (NS), increased costs	€26,962 per QALY. For thresholds at €20 000 the probability of cost effectiveness is 50%
Pyne 2010 [34] USA	Video-link and telephone support for depression N = 335	SF-6D (SF-12) QWB	Baseline, 6 and 12 months	Health provider and patient, comprehensive	QWB 0.015 QALYs gained (NS), SF-6D 0.018 QALYs gained (S), increased costs	$85,634 per QALY (health provider), $132,175 per QALY (incl. patient costs) Not cost-effective
Smith 2008 [35] USA	Monitoring in heart failure N = 1069	SF-6D (SF-36)	Not reported	Health provider	Difference in QALYs not reported, increased costs	$146,870 per QALY, Not cost-effective
van der Meer 2011 [45] Netherland	Internet intervention for asthma N = 200	EQ-5D	Baseline, 3, and 12 months	Societal, comprehensive	0.024 QALYs gained (NS), no difference in costs	$26,700 per QALY, 62% probability of cost-effective at threshold of $50,000
van Keulen 2010 [44] Netherland	Intervention to motivate patients with hypertension N = 1629	SF-6D (SF-36)	Baseline and 7 months	Intervention cost and time costs for the participants	0.02 QALYs gained (S) Telephone most costly	Control group most cost effective for ceiling ratios lower than $2851 per QALY
Van Wier 2012 [46] Netherland	Telephone and e-mail advice for overweight N = 1386	EQ-5D	Baseline, 6, 12, 18 and 24 months	Societal, comprehensive	Phone 0.001 and Internet 0.01 QALYs gained NS, no difference in costs	Internet €1337 per QALY and not cost-effective. Phone €245,000 per QALY. Cost effective at WTP €20,000; 8% for Phone, 60% for Internet and 32% for control
Willems 2007 [29] Netherland	Home monitoring of asthmatics N = 109	SF-6D (SF-36) EQ-5D	Baseline, 4, 8 and 12 months	Societal, comprehensive	Adults 0.03 and children 0.01 QALYs gained (EQ-5D) (S), increased costs (NS)	€31,000 per QALY gained for adults and €59, 000/QALY gained for the children. Limited cost-effectiveness
Yardly 2012 [30] UK	Telephone support for dizziness N = 236	EQ-5D	Baseline, 3 and 12 months	Health provider	0.022 QALYs gained, Increased costs, CI/p not reported	£1363 per QALY, Intervention is cost effective

NS = not significant, S = significant, CI = confidence interval, p = p-value, NMB = net monetary benefit.

*This evaluation study reported mean QALYs gained from 2.45 to 9.44 for 100 individuals.

^§^Intervention group only.

**Table 2:** Publication year

**Year**	**No (%)**
2011 - 2012	7 (40)
2009 - 2010	5 (30)
2007 - 2008	5 (30)

All but one evaluation were conducted alongside prospective randomised controlled trials (RCTs). The one exception analysed home telehealth using a retrospective pre-post evaluation design. It was the only evaluation with cost-effectiveness as a primary outcome measure [25]. Another evaluation used HRQoL as a primary outcome and the EQ-5D as the basis for sample size calculation [26]. In the remaining evaluations, costs and QALYs were secondary outcomes. Sample sizes varied from 48 to 1600. Only four evaluations had less than 200 participants [27-30]. Two evaluations (by the same author) used modelling: one to extrapolate results over ten years [31], and another to map the progress of the participants during the study period [32].

Seven evaluations took a societal perspective on costs and included health care costs, patient costs and production loss. Six evaluations included only health care costs, one included health provider and patient costs and two included only intervention costs. Most evaluations (60%) did a comprehensive cost analysis and included all costs relevant to the reported perspective (see Table 1).

### QALY estimation

All the reviewed evaluations used a validated HRQoL instrument to describe the health states. Two thirds of the evaluations used the EQ-5D and one third used the SF-6D. One evaluation used both EQ-5D and SF-6D and reported results only for EQ-5D utilities [29]. Another evaluation used the HUI3 in combination with the EQ-5D and found more QALYs gained using HUI3 [33]. Another used the QWB in combination with the SF-6D and found a significant improvement only for the SF-6D values [34] (see Table 3). No direct valuation method was used to obtain health state utilities. All the reviewed evaluations collected HRQoL data from patients participating in the actual intervention study. Data were collected at baseline and at regular intervals during the study period. Only one study did not include information about when data had been collected [35]. The study periods varied from 10 weeks to 24 months.

**Table 3:** HRQoL instrument used to obtain QALYs

**HRQoL instruments**	**No. (%)**
EQ-5D	11 (65)
SF-6D	7 (40)
QWB	1 (5)
HUI3	1 (5)

Three evaluations used more than one instrument.

Most evaluations reported the method used to transform the scores from the descriptive systems into utility values. Four used the preference score collected from a sample of the UK population developed by Dolan [36]. Three evaluations used a Dutch preference score developed by Lamers [37,38]. One evaluation mapped the EQ-5D utilities from the SF-12 using an algorithm described by Gray and his colleagues [39]. The algorithm by Brazier et al. was used for SF-6D [18,40]. To transform the QWB into utility values, categorical rating scale values from a community sample and a multi-attribute utility model were used [19]. Hebert et al. [33] estimated QALYs by translating the SF-12 physical and mental score into HUI3 and EQ-5D using a method that has been validated among the African-American patients [41]. Only two evaluations did not report the method used to derive utility values [27,30]. Three evaluations did not report utility estimates [25,35,42].

Two third of the evaluations reported variability around the utility estimates. Half of the evaluations reported baseline and follow-up utility data separately. Five reported adjustment for differences in baseline utility data [27,29,30,33,42]. Most evaluations assumed linear utility changes over time. This was not clearly stated but could be deducted in most cases. QALYs were calculated using the change from baseline score [27,29,31,43,44] or the area under the curve method [33,45,46]; in some cases the calculation was explicitly described [28,42].

### Reporting of results

The mean QALYs gained using telehealth services varied from 0.001 to 0.118 in the reviewed studies. Only six evaluations reported a significant QALY gain [27,29,32-34,44]. All six evaluations reported that the intervention was cost-effective. Three reported that the QALY gain was not significant [43,45,46]. Four evaluations did not report the confidence interval (CI) or p-values [26,28,30,47]. Three evaluations did not report the difference in QALY at all [25,35,42]. In more than half of the evaluations, it was not possible to draw any conclusion about cost-effectiveness on an ordinal level. These evaluations reported small positive differences in QALYs at increased or similar costs but failed to report significance (see Table 1 for details). All, except one [27], calculated incremental cost per QALY or net monetary benefit (NMB).

Five evaluations stated a positive result in favour of telehealth based on thresholds alone [26,27,30,31,47]. Most evaluations calculated the probability of cost-effectiveness within different willingness-to-pay thresholds. Six evaluations reported more than a 60% likelihood of being cost-effective. Two reported a 30% - 50% likelihood of reaching cost-effectiveness. Four reported that the telehealth service was not cost-effective.

Uncertainty due to sampling variation was handled by traditional statistical methods in most evaluations. Three analyses did not include any information on sampling variability in costs and outcomes [25,26,47]. Half of the evaluations (52%) did report CI around the ICER or illustrated the variability in the cost-effectiveness plane. All except three evaluation [25-27], included cost-effectiveness acceptability curves (CEAC). Sensitivity analysis was undertaken in half of the evaluations.

## Discussion

The use of QALYs is recognised as the main valuation technique to measure health outcomes [7,9,24]. Therefore, it is important to consider the appropriateness and transparency of the approaches and methodologies used to estimate QALYs in telehealth studies.

This review identified 17 economic evaluations that used QALYs to measure health outcomes. This seems like a modest number considering that cost-effectiveness is one of the main arguments for telehealth interventions. The number is also quite low, compared to the number of studies that use QALYs in other medical fields. Recent reviews found 33 QALY analyses in spine care [48], 81 studies that used QALYs to measure outcomes in screening programs [6] and 77 evaluations that used QALYs in the field of asthma [49]. However, this review shows that there has been an increased focus on measuring QALYs in telehealth evaluations over the last few years. All 17 studies were published after 2007, and almost half were published in 2011 and 2012.

Most evaluations analysed structured telephone consultations and monitoring of patients at home. More intensive and structured follow-up has been shown to reduce re-hospitalisation and improve patients’ health [50,51]. Only two evaluations included videoconferencing as part of the telehealth intervention. QALYs might be more useful as an outcome measure in studies where the technology is used to provide new or additional services alongside traditional care rather than in studies where videoconferencing is used to replace conventional in-person consultations [3,4].

Most studies originated in the Netherlands, the United Kingdom, the United States and Australia. This might be partially explained by extensive expertise in health economics and the focus on rigorous evaluations before the widespread adoption of any new health care technology or procedure.

The costing methodology has not been considered in detail in this review. However, most evaluations took a health provider and intervention cost perspective. Using the societal perspective in telehealth evaluation is important because it includes costs and benefits for all stakeholders involved, including patient costs associated with travel and treatment [1]. The costs and benefits form a range of different perspectives should be presented alongside a societal perspective [52].

### Preference measure and transparency of the QALY estimation

All the reviewed evaluations used a pre-scored validated HRQoL instrument completed by patients to describe the health states. The evaluations followed accepted methods for transforming the quality scores into utility values. The EQ-5D was the most commonly used method. This coincides with other reviews of QALYs in the literature [53].

One important issue to consider when choosing a preference-based instrument is that each utility instrument is scored based on preferences from a particular population. HUI scores are based on residents of Canada. The EQ-5D and SF-6D use scores based on UK residents. These may not apply to other populations. Only one study used scores validated for a sub-group of the population (African-Americans) [33]. However, several studies have found that when measurements are replicated on different groups of people in different countries, the results are similar [9]. Furthermore, it has been acknowledged that patients tend to give a higher value to health states than the general population [6,54]. None of the reviewed evaluations asked the patients directly to value their health. Asking the patients directly may produce higher utility scores.

The reporting of utility scores at each point in time for each arm of the trial is important for the transparency of QALY estimation, so that the analysis can be replicated. Only half of the evaluations reported baseline and follow-up utility data separately. In most evaluation, it was also unclear whether differences in baseline utility data had been accounted for in the QALY estimation. This implies that the reported QALY gain in these studies can be misleading. Baseline utility is likely to correlate with QALYs and should be accounted for [9]. Manca et al. [55] argued that an imbalance in baseline utility needs to be adjusted regardless of whether these differences are formally statistically significant. They further argued that failure to control for this imbalance can result in misleading incremental cost-effectiveness ratios. Future economic evaluation in telehealth should be transparent in reporting utility data from all time points. They should also control for differences in baseline utility, whether or not these are significant.

Most evaluations reported the variability around the utility measure using relevant statistics. The reporting of the methodology of utility changes over time and the estimation of QALY gain, however, was less convincing. Transparency in reporting the methodology used to calculate QALYs is needed to ensure comparability across telehealth evaluations.

### Reporting of results

The mean QALY gain varied from 0.001 to 0.118 in the reviewed evaluations implying a positive but small effect of telehealth on patient’s health. Only six of these reported that the difference in QALYs were statistically significant. Half of the evaluations did not include a measure of variability around the utility values. Small positive QALY gains have also been found elsewhere. A recent economic evaluation of a large telehealth trial analysed the difference in QALYs for more than 900 patients. It found a small but not significant mean QALY gain of 0.012 [56].

The positive QALY improvements found in this review can contribute to the evidence supporting the claim that telehealth is at least as effective as usual care [57]. However, the absence of a negative QALY effect might be due to publication bias. It could also be because telehealth services with a negative impact have not yet been rigorously evaluated.

Small improvements in utility might not be considered clinically relevant. The minimally clinical important difference (MCID) is defined as the smallest difference in an outcome measure in the domain of interest that is perceived as beneficial [58]. It has been argued that the difference must be at least 0.03 in the utility score to be considered clinically meaningful [59-61]. It has also been demonstrated that the MCID differs between the EQ-5D and SF-6D [62]. Drummond (2001) argued that as long as the ultimate objective is to aid resource allocation decisions, it is the difference in incremental cost per QALY and not the improvement in utility that is important [59]. Most evaluations reviewed in this paper calculated the incremental cost per QALY even if the differences in utility were small and not significant. One of the reviewed evaluations found no significant difference in QALYs and costs but calculated that the telehealth service had a 62% probability of being cost-effective at a threshold of US$50,000 USD [45] (see Table 1).

‘No significant difference’ does not necessarily mean an absence of difference [14]. It can be due to insufficient power, since most economic evaluations alongside trials have clinical measures as primary endpoints. It has also been argued that the difference between two sample means is a better estimate of effect difference than zero [9]. Even if some of the evaluations reported no significant differences in QALYs, none took a cost-minimisation approach. All except one [27] calculated incremental cost per QALY. This is in line with recommended methods. It has also been argued that, if telehealth is going to be adopted on a wider scale, it will have to estimate cost per QALY and pass the same rigorous tests on cost-effectiveness as other new health care interventions [63].

Costs and outcomes of interventions are always associated with some degree of uncertainty. Telehealth is associated with different services, contexts and local settings. Furthermore, parameters such as perspective, measurements, valuation and assumptions regarding cost and outcome identification may affect the results. Uncertainty may be due to sampling variation in cost and outcome data and non-sampling variation related to the economic model and the evaluation process [64]. Assessing uncertainty is important for the validity of the QALY estimation. In these reviewed papers, sampling variation was handled by reporting p-values and CIs for the utility measures. Most evaluations included CIs for the incremental cost per QALY ratio and a quarter illustrated CIs in the cost-effectiveness (CE) plane. To illustrate CIs graphically, cost-effect pairs are plotted in the CE plane, which shows the 95% confidence regions for the ratio [9]. Non-sampling variation is usually handled by sensitivity analyses, which was undertaken in less than half of the studies. This might limit the usefulness of the cost-effectiveness data found in this review as a basis for health care decision making.

Few evaluations in this review stated clear recommendations on the adoption of telehealth. The evaluations used a wide range of affordable thresholds for a QALY. Different countries accept different thresholds. For example, £20,000–£30,000 per QALY has been accepted as the threshold in the United Kingdom, US$50,000–$100,000 per QALY in the United States and AU$76,000 in Australia [65]. Finland, Sweden, Denmark and Belgium do not suggest a threshold value [66]. There might also be differences in the potential value of threshold values for the cost per QALY between different health care systems [67]. These differences should be taken into account when cost-effectiveness results are compared between countries. In practice, whether to adopt new technology may depend on a wider set of objectives than simply to maximise health gain within the budget. These objectives can include the following: to address the lack of alternative treatment options, to reduce the net cost to the health provider, alleviate the burden of the disease, to enhance the innovative nature of the new technology and to address uncertainty regarding cost-effectiveness [68,69].

### Challenges for the QALY approach in telehealth

There are several limitations of using QALYs [70]. One is that the QALY approach does not capture all the benefits of health interventions. Disease-specific measures might capture more benefits than generic HRQoL measures. It has been argued that the EQ-5D and the SF-6D are too generic and insensitive to measure the main outcome of interest for less severe health problems [22,71]. When choosing a utility measure it is important to consider which method is most likely to be sensitive to the health change for the specific patient group included in the study. Disease-specific measures might be more sensitive to the health change that telehealth is likely to produce. Disease-specific measures have been used to calculate QALYs in heart disease and cancer [72,73]. Researchers are working on developing instruments that try to measure broader outcomes within an economic evaluation framework [24].

The benefits of telehealth might extend beyond health outcomes such as access, information, waiting time, time saved and avoidance of burdensome travels. Therefore, for some telehealth interventions, a cost-benefit analysis using the willingness-to-pay approach might be more appropriate.

Another concern is that the QALY model uses different techniques to measure utilities and the results vary according to the method used. Different preference-based instruments can produce different utility values [71]. A number of studies have compared the performance of the SF-6D and the EQ-5D across conditions, settings and patient groups; most of these studies found poor agreement between the utility values [7,74-80]. These two systems vary in several aspects: The SF-6D has more dimensions and levels and explicitly include vitality and functioning. It uses standard gamble to derive utility measures, whereas EQ-5D uses the time-trade-off technique [71,80]. The EQ-5D tends to provide larger change scores and more favourable cost-effectiveness ratios than the SF-6D [77]. One of the reviewed studies used both the EQ-5D and the SF-6D and found positive cost per QALY results for only the EQ-5D utilities [29]. Another used the HUI3 in combination with the EQ-5D and found more QALYs gained using the HUI3 [33]. Ideally, all telehealth studies should use the same utility measure and method. Since different methods have been used in calculating QALYs, results across the telehealth studies should be compared with caution.

### Study limitations

The main purpose of this review was to analyse the methodology and transparency of using QALYs in telehealth evaluations. The scope of this review is therefore quite narrow. Furthermore, excluding economic evaluations that synthesise secondary data in modelling studies is recognised as a limitation. Another limitation is that only articles written in English and published in peer-reviewed journals (to provide basic quality control) were included. In addition, the search strategy used might have overlooked some evaluations. The term ‘telehealth’ is not easily defined; some analysts might have used other terms and definitions to describe remote consultations and the provision of health care over a distance.

## Conclusion

This paper provided a review of the methods used to calculate QALYs in telehealth evaluations. A total of 17 economic evaluations estimating QALYs were identified. All evaluations used validated HRQoL instruments to describe the health states. They also used accepted methods for transforming the quality scores into utility values. The evaluations differed in their choice of methods. Most evaluations reported the methodology used. The evaluations were less transparent in reporting the utility weights at different time points and the variability around utilities and QALYs. The different methods for estimating QALYs and the different threshold values for a QALY may affect the cost-effectiveness results and limit generalisability. It is therefore important to be transparent about the methodology used. Generalisability for telehealth research is problematic in general due to high diversity of technologies used, clinical fields and local health care settings. A more harmonised methodology and utility measure is needed to ensure comparability across telehealth evaluations.

## Competing interests

The author declares no competing interests.

## Authors’ contribution

TSB is the sole author and responsible for conducting the review, analysing the articles and writing the manuscript.

## Pre-publication history

The pre-publication history for this paper can be accessed here:

http://www.biomedcentral.com/1472-6963/14/332/prepub
